# Toxic leadership behaviour of nurse managers and turnover intentions: the mediating role of job satisfaction

**DOI:** 10.1186/s12912-023-01539-8

**Published:** 2023-10-10

**Authors:** Adelaide Maria Ansah Ofei, Collins Atta Poku, Yennuten Paarima, Theresa Barnes, Atswei Adzo Kwashie

**Affiliations:** 1 School of Nursing and Midwifery, University of Ghana, Legon-Accra, Ghana; 2 Department of Nursing, Kwame Nkrumah University of Science and Technology, PMB, University Post Office, Kumasi, Ghana

**Keywords:** Job satisfaction, Nurse managers, Toxic leadership behaviour, Turnover intentions, Ghana

## Abstract

**Introduction:**

Globally, hospitals are confronted with major challenges of turnover of nurses. Knowledge of the factors that account for the turnover of nurses will aid in creating strategies that will enhance nurse managers’ leadership behaviour and job satisfaction to reduce turnover. The study, therefore, investigated the mediating role of job satisfaction on toxic leadership and turnover intentions of nurses.

**Methods:**

A multi-centre cross-sectional study was undertaken to assess 943 nurses using the Toxic-leadership Behaviour of Nurse Managers scale, Minnesota Satisfaction Questionnaire and Turnover Intention scale. Descriptive statistics was used to assess the prevalence of toxic leadership, job satisfaction and turnover and Pearson’s correlation examined the relationships between the variables. Hayes’ PROCESS macro approach of mediation was used to determine the effect of toxic leadership behaviour on the turnover intention on the possible influence of job satisfaction.

**Results:**

The response rate for the study was 76.0%. Mean scores for turnover intentions and toxic leadership behaviour were 3.71 and 2.42 respectively. Nurses who work with toxic managers showed a higher propensity to leave their jobs. Job satisfaction acted as a mediator between the toxic leadership practices of managers and turnover intentions. The total effect of toxic leadership behaviour on turnover intention comprised its direct effect (β = 0.238, SE = 0.017, 95% CI [0.205, 0.271]) and its indirect effect (β = -0.020, SE = 0.017).

**Conclusions:**

Job satisfaction acted as a mediating factor for toxic leadership behaviour and nurses’ turnover intentions. As part of nurse retention initiatives, avoiding toxic leadership behaviours will be the ultimate agenda. Nurse administrators should recognize the value of excellent leadership and develop a structured training programme through the use of evidence-based professional development plans for nurse managers.

## Introduction

The phenomenon of negative leadership in organizations has been labelled in literature as “toxic leadership,“ “dark side of leadership,“ and “abusive leadership” [1–4]. The rise in interest in toxic leadership started when early research found that the failure of leadership is brought on by a variety of causes. Researchers highlighted how the role of the leader’s problematic dispositions impacted the administrative process [5, 6].

Toxic leadership is a term used to describe leaders who exhibit abusive, manipulative, and destructive behaviour towards their subordinates, which can ultimately harm their well-being and the organization’s success. These leaders often prioritize their interests and goals over the needs of their team or organization, and they may use fear, intimidation, or other forms of coercion to achieve their objectives. They also display narcissistic tendencies, meaning they are excessively self-centred and believe they are superior to others. They may be insensitive to the feelings and concerns of their team members and dismiss feedback or criticism from them. They may also create a culture of fear, where employees are afraid to speak up or offer suggestions for fear of retribution [7, 8].

Directing attention to toxic leadership, several researchers have highlighted the role it plays in relationships between leaders and followers, and organizational outcomes. The effects of toxic leadership can be damaging to both individuals and the organization as a whole. While the employees may experience stress, burnout, and mental health issues due to the negative work environment created by the toxic leader, there can also be a cascading result in higher turnover rates, lower productivity, and decreased morale [9–11]. The extreme intoxication of leaders in most work environments has spread a poison of negativity among individuals in organizations, and researchers are concerned about how leaders have lost all sense of purpose and have become so drunk on their power that they are unable to see where this detour is leading organizations [8, 12].

Toxic leadership, for instance, is centred on leaders’ self-interest rather than the collective gain and well-being of followers. Thus, it jeopardizes the “calling” or prosocial essence of the nursing profession. Although inappropriate behaviour by those in leadership or supervisory positions may have significant costs for nurses and patients [13, 14], toxic leadership has not been the topic of considerable research in the nursing field.

According to Ghislieri and Gatti [15], along with Machiavellianism and psychopathy, the so-called “dark triad” of personality traits also includes narcissism (a component of toxic leadership). Toxic leaders create an organizational environment characterized by abuse of authority to manipulate subordinates which may be verbal or non-verbal. It may last until the official association between the manager and the subordinate is terminated or ends, or until the follower modifies their behaviour. Most followers, however, continue to be in toxic relationships because they depend on abusers [16]. It eventually creates serious issues at work, leading to a variety of behaviours like bullying, violence, hostility, and rudeness, among others, which are linked to detrimental psychological effects like diminished self-efficacy [17, 18].

In recent times, nursing professionals are becoming increasingly concerned about the phenomena of abusive leadership in healthcare organizations [19–21], as toxic work culture has become a common issue. In high-income countries (HICs), nurse managers’ (NMs) toxic leadership styles have been reported to have a deleterious impact on the standard of patient care. Working in a toxic environment contributed to nurses’ engaging in unproductive work habits, and expressing a greater desire to quit their job [22]. Many research findings have also emphasized the negative effects of working with a toxic manager on staff and patient well-being, safety, and the overall standard of care [23, 24].

Toxic leadership compromises organization’s ideals and legitimate interests; and these diminish staff morale, self-esteem, and motivation [25]. The organization’s value system is harmed by the toxic environment, making workers less sensitive to others and decreasing employee engagement, with consequences of lack of commitment, job dissatisfaction [26] and turnover intention [27].

Job satisfaction is an essential nursing job outcome, which is influenced by the standard of the workplace. It is a multifaceted phenomenon that describes a pleasant emotional sense resulting from the evaluation of the work or job experience. Staff feel content if their effort produces significant results, which leads to job satisfaction. Additionally, staff who report higher job satisfaction feel good about their jobs, tasks, duties, or work Despite the extensive study on job satisfaction, there are still substantial levels of nurses’ job dissatisfaction [28, 29]. The NM frequently has an impact on the support and motivation that nurses require to perform their duties. Organizations need to recognize the signs of toxic leadership and take steps to address them. This can include providing training and support for leaders to develop more positive leadership styles, holding toxic leaders accountable for their behaviour, and creating a culture of respect and collaboration within the organization. NMs must also establish workplace climates that boost morale and also demonstrate leadership that inspires subordinates. This will eventually increase nurses’ sense of respect, trust, and motivation as well as their level of satisfaction to gradually achieve the organization’s ultimate goal [30, 31].

There is a reported association between nurse job satisfaction and work environment; thus job satisfaction is influenced by the nature of the workplace; the pace, a proportionate workload, relationships with teammates, career opportunities, and the capacity to meet patients’ requirements [32]. To address the significant issues of standardized care, patient outcomes, and nursing job outcomes in healthcare organizations, job satisfaction is essential [33, 34]. It is, however, well acknowledged that effective nursing leadership is the driving force for fostering positive outcomes of job satisfaction and turnover intention.

This concern of turnover is brought up in the context of the workforce, which occurs when individuals leave their jobs or professions because they do not enjoy where they work [35, 36]. Thus, an employee may have a state of mind known as “turnover intention” if they are deeply unhappy at work. The strongest indication for forecasting employee turnover behaviour is typically thought to be turnover intention or intentions to quit [37–39]. From an organizational standpoint, turnover intentions result in financial loss because it causes increased absenteeism and turnover, and affects employee productivity due to a shift in attitude towards their jobs and their well-being [40, 41].

The identified financial impact of turnover on healthcare settings is significant, as most healthcare sectors have used financial and non-financial incentives to encourage nurses to stay on and also recruit new ones, but these initiatives need significant resources and time. Averagely, organizations lose more than US $23 billion as a result of employees quitting their jobs, consequently forcing the closure of facilities; and this is mostly attributed to the toxic workplace [8, 42].

The subject of toxic leadership has become more prevalent and ubiquitous in nursing and healthcare professions in low-middle-income countries (LMICs). Although there is a lot of evidence connecting the toxic environment to poor work outcomes among nurses, the literature has not adequately projected how these behaviours disrupt nursing job processes. The majority of studies, thus, focus on the positive side of leadership and patient and nursing job outcomes [43]. Only a few studies have examined the impact of toxic leadership behaviours on subordinate-leader relationships based on the toxic leadership behaviour taxonomy [44, 45]. In these studies, we have gained a better understanding of how toxic leaders’ maladjusted, malcontent, and malevolent behaviour affects personal and organisational development. Health workers in Ghana, for instance, have complained about poor working conditions and leadership [46], as more than 20% of all nurses have reported the presence of toxic leadership in healthcare facilities [47, 48]. Toxic leadership behaviour of nurse managers has been cited as a contributing factor to nurses’ increased absenteeism and turnover or otherwise [49].

Given the shortage of nurses in LMICs, particularly in sub-Saharan Africa, the lack of adequate information on the influence of toxic leadership on nursing job outcomes such as staff turnover is problematic. It is more significant than ever to assess the impact of toxic leadership behaviour of NMs on turnover intention in the nursing profession or organization as mediated by job satisfaction given the rising number of nurses who want to quit and the high cost of hiring experienced nurses. The outcome of the study may aid in the creation of strategies to enhance nurse managers’ leadership behaviour and job satisfaction, thereby decreasing nurse turnover. The study, therefore, investigated the mediating role of job satisfaction on toxic leadership and turnover intentions of nurses.

## Materials and methods

### Study design and participants

A multi-centre cross-sectional design was used for the study. The design was chosen because it can offer quick evidence for relationships between variables and because researchers were interested in assessing the perception of nurses on toxic leadership behaviour among NMs and their turnover intention. Ghana is divided into 16 administrative regions, each with its unique cultural, geographical, and economic characteristics. These 16 regions are distributed among the three ecological belts in Ghana, including coastal (Greater Accra, Volta, Central, Western and Oti regions), middle (Ashanti, Ahafo, Eastern, Bono, Bono East and Western North regions), and northern (Northern, Savannah, North-East, Upper East and Upper West regions). The study was undertaken in 12 hospitals randomly selected from 6 regions in Ghana. The population of the nursing workforce in the selected hospitals is estimated to be 1716. All participants who had at least a year of working experience in public health facilities were selected as participants.

### Sampling and sample size

A sample size of 1240 participants was calculated using Cochran’s technique [50]. The participants were chosen using a multistage sampling technique. A list of all regions was used as the basis for sampling due to their range of experiences, perspectives, or behaviours to enhance the external validity or generalizability of your findings. Two regions each were randomly selected from each belt (6 regions in all). Two hospitals each were selected from six [6] regions (12 hospitals) to represent the population. Regional hospitals in each of the six regions were purposely selected while a district hospital was randomly selected from the 6 regions. The randomization was done using random number generators. In the 12 selected hospitals, a proportionate stratified sample based on the nursing workforce enrolment was assigned to each hospital. The participants were conveniently selected from the hospitals from each shift within the units. Table 1 presents the population and sample size for each site used for the study.

**Table 1:** Number of Participants by Hospitals (n = 1240)

Hospitals	Population of nurses	Calculated (expected) sample size	Response rate (% of sample size)	% of the total population in the study	
Site 1	238	172	138 (80.2%)	14.6	
Site 2	194	140	92 (65.7%)	9.8	
Site 3	181	131	95 (72.5%)	10.1	
Site 4	125	90	71 (78.9%)	7.5	
Site 5	146	106	83 (78.3%)	8.7	
Site 6	115	83	64 (77.1%)	6.8	
Site 7	152	110	75 (68.2%)	8.0	
Site 8	104	75	61 (81.3%)	6.5	
Site 9	162	117	98 (83.8%)	10.4	
Site 10	107	77	62 (80.5%)	6.6	
Site 11	91	66	48 (72.7%)	5.1	
Site 12	101	73	56 (76.7%)	5.9	
**Total**	**1716**	**1240**	**943 (76.0%)**	**100**	

### Method of data collection

Formal and ethical approvals were sought from the various hospitals’ management and the review board respectively. Communication with participants was established and data collection was initiated with the assistance of the NMs and the ward in-charge from September to December 2021. The questionnaire was in English language and the researchers administered it themselves. The study’s participants voluntarily joined the study, and they were made aware of their right to withdraw from the study. The study’s potential benefits and risks were also discussed before participants signed an informed written consent form. The researchers collected the completed questionnaires from the participants.

### Measures

The Turnover Intention (TIS-6), Minnesota Satisfaction Scale (MSQ) and the Toxic Leadership Behaviours of Nurse Managers’ Scale (ToxBH-NM) were the three self-report scales used.

#### Toxic leadership behaviour of nurse managers

The ToxBH-NM scale with a 30-item was used [51]. The scale has four sub-dimensions of toxic leadership - humiliating behaviour (3 items), intemperate behaviour (15 items), narcissistic behaviour (9 items), and self-promoting behaviour (3 items) was used to measure nurses’ perceptions of the toxic leadership behaviours of NMs. A Likert scale with five possible ratings was used (1 - not at all and 5 - All the time). Non-toxic (1.0–2.2 points), moderately toxic (2.3–3.6 points), and highly toxic (3.7–5.0 points) were the interpretations of the ToxBH-NM composite score. When the sub-scale composite mean is higher, it signifies that toxic leadership behaviour is more prevalent. The Cronbach alpha for the ToxBH-NM scale as reported was 0.88. In previous studies, the scale also indicated a satisfactory reliability coefficient score of at least 0.70 [2, 52, 53].

#### Job satisfaction of nurses

Nurses’ job satisfaction was assessed using the Minnesota Satisfaction Questionnaire (MSQ-short version) [54]. The scale had 20 items and a Likert-type scale with a range of 1 to 5 (very dissatisfied-1 and very satisfied-5). The composite score of all items is divided into 1.0–3.0 (low satisfaction), 3.1-4.0 (moderate satisfaction, and 4.1-5.0 (high satisfaction). Satisfactory reliability coefficients range from 0.85 to 0.91 in previous research that employed this scale [55–58].

#### Turnover intentions

Nurses’ intention to quit their current job and/or profession was assessed using a 6-item TIS [59]. The scale is measured with a Likert scale between 1 = Never to 7 = Most of the time. A composite mean score of ≥ 3.5 indicated a higher turnover intention of the nurse. The Reliability Coefficient of the scale was 0.80 [60].

### Data analysis

Using SPSS software version 26, descriptive and inferential statistics were used to analyze the data. While means, percentages, and standard deviations were used to describe the data, Pearson Moment Correlation was used to assess the relationship between the variables. Mediation analysis was also undertaken using Hayes’ PROCESS macro (Model 4, version 4.2) to examine the impact of toxic leadership behaviour and job satisfaction on turnover intentions. Hayes’s mediation approach is a powerful analytical method that has been recommended for nursing research [61, 62]. It is a percentile bootstrapping process whereby the sample distribution is resampled 5000 times to calculate mediation effects at a 95% Confidence Interval (CI). Statistically significant variables in univariate analysis were included as covariates (age, highest qualification and duration at the facility): Toxic leadership behaviour as an independent variable (X); job satisfaction as a mediator variable (M); and turnover intention as a dependent variable (Y). The macro allows calculating and testing the direct effect, the total effect, and the indirect effect. The indirect effect is considered statistically significant if zero is not included in the reported CI. All study variables were tested for multicollinearity and a *p*-value < 0.05 was considered statistically significant.

### Ethical considerations

According to the Helsinki Declaration, ethics was sought from the Noguchi Memorial Institute of Medical Research of the University of Ghana (NMIMR-IRB CPN 010/21–22). Before administering the questionnaire, written informed consent was requested from all participants; confidentiality and anonymity were also ensured. Participants were made aware of their right to withdraw in the course of the study when the need be.

## Results

### Demographic data and nurses’ job characteristics

With a sample size of 1240, a total of 943 fully answered questionnaires were recovered from participants (76.0% response rate). The mean age of the participants was 30 (SD:4.43) years. Moreover, half of the participants (n = 267, 57.8%) were single and women made up more than two-thirds of the participant population (n = 678, 71.9%). Close to a third of the participants (n = 286, or 30.3%) constituted staff nurses, and nearly half (n = 487, or 51.7%), and 50.5% (n = 469) had worked at their current facility for at least five years. Participants’ sociodemographic and job characteristics details are shown in Table 2.

**Table 2:** Socio-demographic characteristics of the participants

Socio-demographic data	N=943	%	Mean	SD
Age			30	4.43
Gender				
Male	265	28.1		
Female	678	71.9		
Marital status				
Married	398	42.2		
Unmarried	545	57.8		
Highest qualification				
Certificate	263	27.9		
Diploma	487	51.7		
Degree	172	18.2		
Masters	21	2.2		
Rank				
Enrolled Nurse (Snr/Prin. EN)	269	28.5		
Staff Nurse/Snr Staff Nurse	486	51.6		
Nursing Officer	121	21.8		
Senior Nursing Officer	67	7.1		
Duration at facility				
Less than 2 years	108	11.4		
2–4 years	359	38.1		
4 years and more	469	50.5		

### Toxic leadership behaviour, job satisfaction and turnover intentions

The results of the perception of toxic leadership behaviours among NMs, job satisfaction, and nurses’ turnover intention are shown in Table 3. The mean score for toxic leadership behaviour of NMs was 2.42 (SD: 1.39); the subscale measuring narcissistic behaviour had a composite mean score of 2.53 (SD: 1.36) followed by humiliating behaviour (M: 2.49, SD: 1.45), Intemperate behaviour (M: 2.40, SD: 1.36), and self-promoting behaviour (M: 2.27, SD: 1.38). The composite mean score of nurses’ job satisfaction and turnover intentions were 2.75 (SD = 0.99) and 4.71 (SD = 1.56) respectively.

**Table 3:** Toxic Leadership Behaviour, Job Satisfaction and Turnover Intention of Nurses

Scale/Subscales		Composite Mean	SD
Toxic Leadership behaviour		2.42	1.39
	Intemperate behaviour	2.40	1.35
	Narcissistic behaviour	2.53	1.36
	Self-promoting behaviour	2.27	1.38
Job Satisfaction Turnover Intention	Humiliating behaviour	2.49 2.75 4.71	1.45 0.99 1.56

### Relationship between job satisfaction, turnover intentions and perception of toxic leadership behaviour of NMs

Using Pearson’s moment product correlation, the association between the toxic leadership behaviour of NMs, turnover intentions, and job satisfaction were measured as presented in Table 4. A significant positive relationship was established between the turnover intention of nurses and scores on the various dimensions of toxic leadership behaviour; narcissistic behaviour (*r* = .383, *p* < .01), self-promoting behaviour (*r* = .483, *p* < .01), humiliating behaviour (*r* = .336, *p* < .01), intemperate behaviour (*r* = .368, *p* < .01), and toxic leadership behaviour (*r* = .406, *p* < .01). A negative significant correlation was noted between job satisfaction and turnover intentions (*r* = − .146, *p* < .01) and toxic leadership behaviour (*r* = − .517, *p* < .01).

**Table 4:** Correlation between Toxic Leadership Behaviour, Turnover Intentions, and Job Satisfaction

Correlations	1	2	3	4	5	6	7
1. Turnover Intentions	1						
2. Job Satisfaction	− .146^*^	1					
3. Narcissistic Behaviour	.383^*^	− .490^*^	1				
4. Self-Promoting Behaviour	.483^*^	− .139^*^	.594^*^	1			
5. Humiliating Behaviour	.336^*^	− .481^*^	.879^*^	.575^*^	1		
6. Intemperate behaviour	.368^*^	− .551^*^	.936^*^	.515^*^	.863^*^	1	
7. Toxic Leadership Behaviour	.406^*^	− .517^*^	.977^*^	.634^*^	.911^*^	.980^*^	1

^*^ *p < .01*

### Impact of toxic leadership behaviour and job satisfaction on turnover intention

**Fig. 1:**
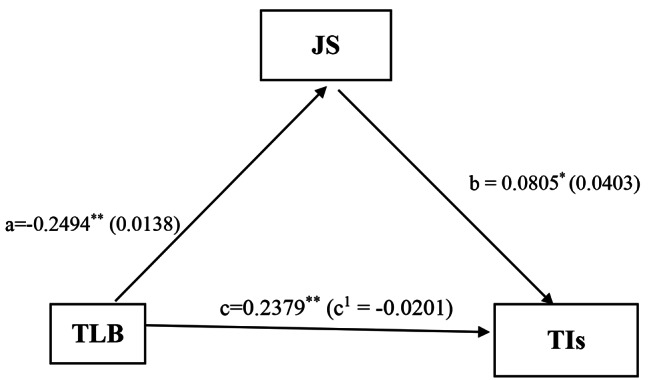
The impact of toxic leadership behaviour on turnover intentions: the mediating role of job satisfaction Mediation model explaining the relationship between Toxic Leadership Behaviour (TLB) and Turnover Intentions (TIs) among nurses through Job Satisfaction (JS) (in Table 5). N = 943; controlled for age, highest qualification and duration at the facility; a = direct effect of TLB on JS; b = direct effect of JS on TIs; c = total effect of TLB on TIs; c^1^ = direct effect of TLB on TIs. *p < .05, **p < .001

The mediation role of toxic leadership behaviour and job satisfaction on turnover intention is detailed in Table 5. Toxic leadership behaviour had a significantly negative association with job satisfaction (estimate for *a* = -0.2494, SE = 0.0138, 95% CI [-0.2799 to -0.2223]). Also, job satisfaction had a positive association with turnover intention (estimate for *c* = 0.0805, SE = 0.0403, 95% CI [0.0014 to 0.1595]). The total indirect effect of toxic leadership behaviour on turnover intentions was statistically significant (*b*= -0.0201, *SE* = 0.0114, 95% CI [-0.0436 to -0.0019]. The bootstrapped CI for the indirect effect was below zero, suggesting a statistically significant mediation effect. The model (Fig. 1) demonstrated that the negative relationship between toxic leadership behaviour and turnover intention was statistically significantly mediated by job satisfaction (estimate for *b* = 0.2379, SE = 0.0170, 95% CI [0.2046 to 0.2712]).

**Table 5:** Results of Mediation analysis

Paths	Estimate	SE	t	p-value	95% CI	R^2^
TLB→JS	− 0.2494	0.0138	-18.0637	0.0000	(-0.2766, -0.2223)	0.5267
JS→TIs	0.0805	0.0403	1.9967	0.0462	(0.0014, 0.1595)	0.4455
TL→TIs	0.2379	0.0170	14.0348	0.0000	(0.2046, 0.2712)	0.4416
Indirect effect	Effect	Boot SE	Boot LLCI	Boot ULCI		
TLB→JS→Tis	− 0.0201	0.0114	− 0.0436	− 0.0019		

Toxic Leadership Behaviour; TLB, Turnover Intentions; TIs, Job Satisfaction; JS, bootstrap standard error; Boot SE, variance accounted for; R^2^, lower limit confidence interval; LLCI, standard error; *SE*, upper limit confidence interval; ULCI

## Discussion

This study augments the corpus of data on NMs’ toxic leadership and nurses’ turnover intention in Ghana. This study assesses the role of toxic leadership on turnover intention among staff nurses as mediated by job satisfaction. Nurses’ job satisfaction declines as toxic leadership behaviour occurs more frequently. The turnover intention (to leave the profession or organization) follows a decline in job satisfaction. It has also been empirically proven that this link exists [63–65]. Meanwhile, the study reported a higher turnover intention among nurses; and is consistent with a study among nurses in China [63], the US [66, 67], Saudi Arabia [68] and the Philippines [69]. A possible explanation for this phenomenon is the rise in social demand for nurses. Nurses require greater attention if they are to have lesser intentions of leaving their jobs.

This study finding is consistent with what was reported in Egypt with nurses perceiving their managers as moderately toxic [70], though low to no toxic leadership behaviour has been reported in parts of the world [52, 71]. These findings on the specific predictors of turnover intentions should provide helpful information for hospital and nursing leaders when implementing interventions to improve job satisfaction and retain nurses. This is a disappointing conclusion given the relative role of leadership in creating and promoting nursing work environments that empower nurses and advance nursing job and patient outcomes [52, 72, 73].

Additionally, nurses have a moderately negative perception of the toxic leadership behaviours of NMs, suggesting that nurse managers are likely to engage in such behaviours. Conspicuously, the mean score of all subscales falls into the moderate category. The results are consistent with other studies that noted NM’s toxic leadership behaviour to be poor [74, 75]. This outcome is significant in light of the negative effects that having NMs who practice toxic leadership can have on the organization and its staff.

The significant consequence of toxic leadership of NMs on nursing job outcomes, particularly, turnover intentions was perhaps the most important finding of this study. Nurses who work with NMs who exhibited toxic leadership behaviours expressed a greater desire to quit their jobs and or even their profession. A similar position was reported in Canada by Lavoie-Tremblay et al. [22] who reported that nurses indicated a higher turnover intention when they observed more toxic leadership practices. By establishing a connection between toxic leadership behaviours and nurses’ intentions to leave the profession through the lens of job satisfaction, this study adds new knowledge to the field of nursing, principally in the areas of nursing management.

The negative impacts of toxic leadership behaviours by NMs on nurses’ work attitudes were more or less anticipated and consistent with the literature. Intemperate, humiliating, narcissistic, and self-promotion behaviour are frequently used by toxic leaders, which results in job dissatisfaction, productivity as well as a lack of motivation for their jobs [34], frequent absenteeism [76], and increased turnover intentions [77]. This finding supported earlier research showing that a toxic leader’s actions and behaviours, which were primarily motivated by personal interests to advance their growth and advancement, had a significant negative impact on nurses’ level of job satisfaction, which ultimately led to turnover [78–80].

Accordingly, there was a positive correlation between toxic leadership behaviours and intentions to leave the organization. As a way to reduce turnover, a potential institutional measure in the form of a development plan for effective leadership practices among NMs through training, and policy formulation should be instituted to lessen the incidence of toxic leadership behaviours.

A considerable amount of research has been done on the level of job satisfaction among nurses in several countries, although further studies are needed in West Africa and Ghana. According to our study, nurse job satisfaction is low in Ghana. We found that our results were almost similar to what we found among nurses in Ethiopia [81], Kenya [82] and Ghana [83]. In Ghana, however, job satisfaction among nurses has been studied extensively, but not concerning toxic leadership behaviour.

Job satisfaction acts as a buffer against the negative effects of toxic leadership on nurses’ turnover intentions. Several studies have shown that toxic leadership behaviour can have a significant impact on nurses’ job satisfaction and turnover intentions. Toxic leaders can create a negative work environment that causes stress, burnout, and low morale among nurses, and in turn, can lead to reduced job satisfaction and increased turnover intentions [27, 52, 84]. When nurses are satisfied with their jobs, they are more likely to be committed to their organizations, have higher levels of productivity, and experience less stress and burnout. Consequently, job satisfaction can mediate the relationship between toxic leadership behaviour and turnover intentions. When nurses are satisfied with their jobs, they may be less likely to leave their jobs, even in the face of toxic leadership behaviour. Conversely, when nurses are dissatisfied with their jobs, they may be more likely to leave, even in the absence of toxic leadership behaviour. The mediation of job satisfaction on the relationship between toxic leadership behaviour of managers and turnover intentions of nurses, therefore, highlights the importance of creating a positive work environment that supports nurses’ job satisfaction and retention. Organizations can do this by promoting positive leadership behaviours, providing opportunities for professional development and growth, and fostering a culture of respect, trust, and collaboration [34, 85, 86].

### Implications

The finding of the study is an indication that organizational measures to overcome nurse turnover should include tackling toxic leadership practices. To reduce or avoid toxic behaviours among NMs, some of the most important interventions are education, training, and professional development. The structured leadership development training modules should be a priority of every healthcare organization. The training will help nurse managers to acquire the skills and knowledge needed to lead effectively. This may include training in communication, conflict resolution, emotional intelligence, and other key leadership competencies.

Again, organizations should foster a positive organizational culture by promoting values such as respect, trust, and transparency. When these values are ingrained in the culture of an organization, it is less likely that toxic leadership behaviour will be tolerated or encouraged. Nurse leaders can effectively build a positive workplace culture by staying up to date on the most recent research on effective leadership practices. Moreover, healthcare managers must encourage open communication by creating channels for employees to provide feedback and express their concerns. When employees feel that their voices are being heard and their opinions are valued, they are more likely to report toxic behaviour and seek help when needed. Every organization should make it standard practice to have a zero-tolerance policy intended to reduce toxic behaviours at work and a clear policy that sets behaviour expectations for all staff. Therefore, a leadership assessment tool may be used by recruitment teams tasked with finding competent NM candidates to screen and identify leaders who can help the organization accomplish its goals. By gathering feedback from the members of the health team, a leader evaluation utilizing a suitable method may help understand the leader’s performance and leadership needs. Programs such as mentoring and coaching for new NMs may also be beneficial.

Additionally, the improvement of NMs’ leadership behaviour and the advancement of their professional growth may be possible through the pursuit of higher education and the acquisition of essential training.

Training courses for learning healthy ways to manage emotions should be encouraged to effectively avoid exhibiting toxic behaviours. The construction and development of strong leadership styles in the future nursing workforce depend on nurse education at the graduate level, which emphasizes the need for successful leadership as well as techniques to improve leadership competencies. Not only does toxic leadership behaviour impact on turnover of nurses, but it also has negative effects on the quality of care and patient safety [23, 32, 87].

### Limitations

The methodology utilized in this study makes it difficult to establish causality. By examining nurses’ perceptions of their leaders, we evaluated the toxic leadership practices of NMs. Based on the type of relationship between the NMs and their subordinates, this strategy may present a socially desired response. However, the likelihood of obtaining skewed responses was decreased by using a multi-stage sampling technique and a sizable sample (n = 943). Although nurses’ views about the toxic leadership behaviour of NMs may significantly influence turnover intentions, other factors such as the workplace environment, the presence of collegiate nurse-physicians relation, the adequacy of resources, and nurses’ participation in decision-making may also be possible mediators; future studies should therefore focus on employing other designs such as mixed-method or observational designs to comprehensively analyse the leadership behaviour of NMs and other associated characteristics that affect the nursing workforce. Once the baseline data from this study has been used to suggest future directions, investigating other detrimental contributions of toxic leadership by NMs, such as absenteeism, workplace violence, incivility, and adverse patient outcomes should be studied.

## Conclusion

The study has produced new insight into nursing leadership and administration. In terms of their leadership styles, nurses often evaluated their NMs as being moderately toxic. This position supports earlier studies that found a link between the toxic leadership behaviour of NMs and poor nursing job outcomes, especially, the turnover intention of leaving the profession or post.

Nurse managers’ toxic leadership behaviour harms nurse turnover intentions, mediated by job satisfaction, underscoring the critical importance of addressing leadership dynamics within healthcare organizations. To ensure nurse retention and improve the overall quality of patient care, healthcare institutions must cultivate a positive and supportive leadership culture that promotes job satisfaction. It is important that toxic leadership behaviours are addressed and that a nurturing work environment is promoted as part of healthcare management and leadership strategies. This can be achieved through training on professional development and implementation of leadership reform strategies to derail toxic leadership behaviours among NMs.

## Data Availability

All data generated or analyzed during this study are included in this published article.

## List of abbreviations

HICs: High-income countries
JS: Job Satisfaction
LMICs: Low-middle-income countries
MSQ: Minnesota Satisfaction Questionnaire
NMs: Nurse Managers
TIS: Turnover Intention Scale
ToxBH: NM-Toxic Leadership Behaviour of Nurse Managers
